# Orthodontia

**Published:** 1866-07

**Authors:** A. A. Blount


### "ORTHODONTIA."

BY A. A. BLOUNT, D. D. S.

Read before the Mad River Dental Society.

Having, in the last few years, given the subject of orthodontia a degree of attention commensurate with its importance, I feel that I can speak with a degree of confidence of the result of my experience, and not, as is too often the case, treat it theoretically.

There are so few who give this subject the consideration its importance demands, that it should be brought more prominently before the profession; and there is no better way to effect this than to make it a subject of frequent discussion at our dental meetings. The excuse offered by many who are unwilling to undertake the treatment of cases of irregularity, is that it is non-compensating. This is undoubtedly true in a great many instances; but, an the other hand, we have the satisfaction, when we meet our patient, of contemplating a perfect denture. Nothing is so repulsive as an imperfect and irregular set of teeth.

The remote or obscure causes which produce irregularity, will be found in the "commingling of all nations, with their national and individual peculiarities," of which material the American nation is composed. Is it to be wondered at, then, that America is the Paradise of the dentist? Not only does this commingling, in a great measure, produce dental caries, but also dental irregularities, as well as other deformities. The child will, as a general thing, inherit the form of teeth and mouth of the parent, whom it most resembles in other respects; occasionally we will find the mouths of both parents reproduced in that of the child—it will inherit from one a small contracted dental arch, and from the other, large well developed teeth, which are so distorted and crowded together that the dentist will despair of ever treating the case successfully, without resorting to a wholesale extraction.

The most frequent causes which produce irregularity are comprised in the following: The result of accident, the indiscriminate extraction of the decidious, and the too early extraction of the permanent teeth. Of these causes, the most disastrous is the loss of deciduous teeth,—there is nothing after which a train of evil results follow so surely and rapidly as the premature extraction of these teeth.

Allow me, in this connection, to make a few extracts from an English author of note, and observe where his teachings, if followed out, would lead. I fear there are many, even now, who follow him to the very letter. In an article on second dentition, he says, "In most cases it will be proper to remove the four temporary incisors, as the two central permanent teeth advance, to allow them to take a position to form a circle of sufficient scope to allow of their *regular* arrangement." He argues, that, retaining the two temporary laterals does not contribute to the expansion of the jaw, and therefore, they are only in the way. He extracts two more temporary, as the two lateral permanent teeth appear. Thus he has extracted six decidious teeth, for the four permanent. So he goes on until the permanent teeth are all erupted, except the two cuspidati, which are now making their appearance in front of the arch. He says, "room, in such cases, must of course be made for them, at whatever expense," and then goes on to say, "that the first permanent molars, erupted at an early age, when the child was physically weak, were almost always found decayed, or with defective enamel, so as to present early signs of decay, thus nature points out, at once, the remedy, extraction of these teeth."

This practice not only does mischief in the removal of decidious teeth alone, but also in the extraction of the six year molars. There is no deformity so destructive to the beauty and symmetry of the mouth, and none so difficult to correct, as that resulting from the early loss of these teeth. How many otherwise, beautiful and expressive mouths have been rendered repulsive in their expression, by this deplorable practice!

We are, at this present time, treating two cases of malposition of the teeth and jaws, both of which are the result of the early extraction of the decidious and permanent molars. In one case the two six year molars in the inferior maxillary were removed at an early age; and the ten anterior teeth were drawn back by the pressure of the lip, which so shortened the lower arch as to give a very disagreeable expression to the under lip, it being turned out by closing upon the superior incisors. The other case is that of a lovely little girl of twelve or thirteen years, whose inferior incisors and cuspids, close in front of the superior incisors, throwing the chin forward, giving it an unnatural prominence, also a contraction of the angles of the jaw, making what is termed a "rabbit mouth." This deformity was caused by the premature extraction of the decidious molars, allowing the six year molars, and first bicuspids, to close upon each other, leaving no room for the second bicuspids to come in, which, consequently never made their appearance. It is scarcely necessary to mention the various forms of irregularity, as almost every case presents a different condition. We will mention the fact, however, that irregularity occurs most frequently in those teeth anterior to the first molars. During the eruption of the permanent teeth, nature displays great wisdom in the manner in which she proceeds to enlarge the jaw sufficiently to allow all the permanent teeth to take a proper position.

In the first place, the two central incisors make their appearance almost as if not fully double the size of the decidious incisors, it would seem almost impossible for them to obtain sufficient room, but by a gradual and constant lateral pressure, space is soon obtained. In a little while, the laterals begin to crowd their way down. Sometimes they do not come exactly between the decidious eye-teeth and permanent centrals. Therefore, instead of producing the lateral pressure requisite to expand the jaw, they are twisted around, so as to present the least resistance to their outward growth, and consequently present a very crowded appearance. In many instances, at this period, the child is taken to the dentist, who is urged, by the anxious parents, to remove the temporary eye-teeth to make room for the crowded laterals. In a short time, the first bicuspids make their appearance, and seem to enhance the difficulty by pushing forward the decidious eye-teeth. The dentist is again importuned by the parent to extract the temporary cuspids. But should he remain firm, and resist the entreaties, and sometimes threats of the parent, he will soon have the satisfaction of seeing the jaw begin to expand, the second bicuspids coming into place, and the cuspids gradually approaching their proper position in the arch, to complete its beauty. Thus nature, by seeming to hurry the teeth forward too rapidly, accomplishes the end desired at the proper time, expanding the jaw sufficiently to allow the teeth to assume their position in the dental arch.

In treating orthodontia, almost every dentist has his own peculiar ideas, and a different mode of constructing the appliance. They may all differ in their construction, yet all possess the same general principles.

In a large majority of cases of irregularity, in quite young persons, it is better not to attempt a treatment, but to allow nature to make such changes as she will, until such time as the jaw has attained nearly its full size. In many instances she will have accomplished the task as skillfully, if not more so, than if done by the dentist. When left to herself, and uncontrolled by hereditary influence, nature will make every portion of the body perfect, in form and structure.

It is always desirable, and *imperative*, in young persons, to obtain the results in regulating, without resorting to extraction to make room. The only instances where extraction is admissible, is in patients of such an advanced age, that it would be difficult, and even dangerous, to attempt moving too many teeth at one time. It will often be found necessary, in order to straighten a single tooth, to change the position of several of the adjacent teeth. We are treating a case at present, in which, to regulate óne tooth, we are compelled to move five of the adjoining teeth, in order to make them all conform to the altered position of the crooked one. But should it become absolutely necessary to extract, let it be done with caution, and after mature reflection. It requires experience and good common sense, to discriminate in all cases. I have seen many beautiful and otherwise perfect dental arches completely ruined, by a thoughtless or careless extraction of a single tooth. An instance of recent date, of a young lady of my acquaintance, who had a cuspid standing in front of the arch, which could have been readily drawn into place, but, while on a visit to a neighboring city, went with a friend to one of the first dentists, who, be it said to his shame, extracted the eye-tooth. That lady never looks into her mouth, but to lament the loss of that tooth.

Since the introduction of vulcanized rubber into mechanical dentistry, our facilities for constructing appliances have been greatly enhanced. The difficulties which heretofore attended the manufacture of regulating plates have been entirely overcome. The ease and facility with which rubber is adapted to the various purposes of this kind, render it invaluable to the dentist.

It is hardly necessary to go into a minute and detailed description of various methods of constructing appliances for the different forms of irregularity, as every case, when thoroughly studied, will suggest to the mind of the operator, the means of overcoming it. If we desire to press a tooth or teeth out, which close inside of the inferior, or superior arch, we construct a plate of rubber, allowing caps to extend over the molars to prevent the teeth from coming in contact, build up behind each tooth it is desired to move, an upright of rubber, sufficiently strong to admit of considerable pressure. Into this upright, a screw can be placed to bear upon the tooth, or a stout piece of elastic rubber may be drawn through a hole, drilled in the upright, at a point desired to produce the pressure, which can be regulated at the will of the operator or patient. If the teeth stand in front of the arch, and we wish to draw them in, and at the same time expand the angles of the jaw, construct uprights from the angles back to the molars, vulcanize a piece of platinum or gold wire in the plate at the point we desire the teeth to be drawn, bend the wire down in the form of a hook, on which to fasten a piece of elastic tubeing, to be drawn forward over the tooth to be moved. In this manner, all the force desired can be obtained. Between the other teeth and uprights pack cotton, or fasten rubber, as before described.

These are the only means I have ever found necessary to resort to, to accomplish the object in the most difficult case.

The more simple and adjustable the appliance is made, the better it will answer the purpose, and the more easily will it be worn. Young people have not much patience to bear pain, and soon become tired of anything that requires constant care and attention. In all cases it is advisable not to move the teeth too rapidly at the commencement, but let the pressure be gentle, till the bony obstructions begin to yield, when the soreness will be much less than in the beginning, and the case can be hurried forward more rapidly to completion.

I do not know that I have advanced a single idea that is new, or that, what I have written will afford any member of this society a particle of instruction; but I was appointed to write an essay, and chose this subject, because it is one in which I am deeply interested. If every member would choose a subject in which he feels an interest, and write upon it, we would be benefitted and instructed as well as himself.

